# Editorial Comment

**Published:** 1952-01

**Authors:** 


### Editorial Comment

For nearly thirty years Mr. Eric Watson-Williams has served this *Journal*, as reporter, assistant editor, and editor. Without his zeal and devotion through the years it is likely that it would long since have ceased to exist. The *Medico-Chirurgical Society* owes to Mr. Watson-Williams a deep debt of gratitude and it is fitting that on his retirement from the editorial chair he has been elected President of the Society for the next year.

Dr. Nicholas Craig who since 1948 has undertaken the thankless task of assistant editor, feels that he is not sufficiently in contact with his medical colleagues in the University and Hospitals to continue the functions of that post to his own satisfaction, and has resigned. Our readers will wish us to acknowledge with sincere thanks all the work he has done on behalf of the *Journal.*

### EDITORIAL POLICY

This issue appears under the guidance of a new Editorial Committee. We wish to welcome Dr. J. E. Cates, Dr. A. H. Gale, and Dr. A. M. MacLachlan as new members, and to thank Dr. Sutton, business manager and Dr. Middlemiss for their continued interest. The *Journal*, as seems to be its habit with a change of editor, has acquired a new garb, but its policy remains that of a medical journal for the south-west.

The 1946 Act divided the country into medical regions which were thought to be large enough to make each a homogeneous unit and small enough to enable all the medical services in the region to be closely integrated. These objects are still far from achievement, and to some it seems that the four big divisions of the medical services: public health, general practice, the hospital service and the administration are moving further apart rather than closer together. A letter in this issue from Dr. Woolley exemplifies the problems which face us. We all wish to know each other's work better but no satisfactory basis of co-ordination has appeared.

It is hoped that this *Journal* in striving to continue its function as a mirror of medical activities in the South-West Region may play a part in strengthening the bonds between the ever increasing branches of medicine, clinical, administrative and social. We ask for articles, letters, comment and criticism from all who are interested in medicine in the region. Contributions to the correspondence columns are very welcome.

### AFTERMATH OF POLIOMYELITIS

Elsewhere in this issue Dr. Macrae has discussed the immediate effects of the epidemic of poliomyelitis of 1950 and it may be of interest to our readers to know something of the subsequent history of the 316 patients up to the time of going to press, 1st December, 1951. Two more deaths have occurred in Winford Hospital making thirty-four in all. Fourteen patients are still in hospital and the rest have been discharged to their homes. We hope to include a more detailed account of the subsequent progress of these patients in a later issue.

A report on the International Congress in Copenhagen by Dr. Macrae also appears elsewhere in this issue. Dr. Macrae and Dr. D. F. Johnstone attended this conference as representatives of the Regional Hospital Board.

### A MEDICAL CASUALTY

Members of the Medico-Chirurgical Society, and his many friends in the south west, will have heard with regret the serious illness of Mr. A. J. Wright, following a road accident. As we go to press we are glad to learn that he has sufficiently recovered to be able to go home. Mr. A. J. Wright retired from practice as an E.N.T. specialist at the end of the war, when he became the first Director of Post-Graduate Studies at Bristol University. He entered the City Council for the Clifton Ward in 1943, and is now Chairman of the Health Committee; he is also Chairman of Frenchay Hospital. We wish him a speedy and complete recovery.

### HOSPITAL SITING

Mr. Wilfrid Adams after a major operation and a year on the sick list celebrated his recovery by delivering a Hunterian Lecture to the College of Surgeons on *The Range and Practice of Transur thral Surgery*, and has returned to work with renewed vigour.

He has stimulated much interest by raising for discussion the future siting of the Bristol teaching hospital. Present plans provide for the building of a new United Bristol Hospital on the area between Upper Maudlin Street and St. Michael's Hill. This site is central, near the other hospital services and close to the University with its pre-clinical departments.

Mr. Adams proposed that the Board of Governors should ask the Local Planning Authorities, Bristol City Council and Somerset County Council, to reserve one hundred acres of Ashton Court as an optional alternative site on which the teaching hospital for the Region might be developed if medical opinion favoured a peripheral position, when the time comes to rebuild. In favour of such a move are the spaciousness and pleasant surroundings in which the architects might be expected to plan an attractive and well-proportioned hospital while allowing space for future developments; the avoidance of the traffic congestion in the St. Michael's Hill area which is likely to become more acute as more cars come on the road; and the provision of accommodation for the long-stay patients to provide a comprehensive cross section of the hospital sick, of which students would get a more balanced view.

In either case the provision of a general practice block or wards within the teaching hospital would probably do more than any other single move to improve the collaboration between general practitioners and specialists.

Though Mr. Adams's proposal was approved by the Medical Committee, the Board of Governors has decided against any change of policy at the present time. The Local Authorities are obliged to review their planning allocations every five years so that an opportunity will arise for a further examination of the proposals in 1956. As there seems no prospect of any new hospital building within ten years there is time for much thought and discussion before a final decision is reached.

